# Supplementary material for: Effects of Substrates on Nucleation, Growth and Electrical Property of Vertical Few-Layer Graphene
Source: Nanomaterials (Basel). 2022 Mar 15;12(6):971. doi: 10.3390/nano12060971 (PMC8950384; doi:10.3390/nano12060971)
Supplement: Supplementary file 1 [file nanomaterials-12-00971-s001.zip › nanomaterials-1611554-supplementary.pdf]

Effects of Substrates on Nucleation, Growth and Electrical Property of Vertical Few-Layer Graphene

Tianzeng Hong, Chan Guo, Yu Zhang *, Runze Zhan, Peng Zhao, Baohong Li and Shaozhi Deng *

State Key Laboratory of Optoelectronic Materials and Technologies, Guangdong Province Key Laboratory of Display Material and Technology, School of Electronics and Information Technology, Sun Yat-sen University, Guangzhou 510275, China; hongtz@mail2.sysu.edu.cn (T.H.); guoch5@mail2.sysu.edu.cn (C.G.); zhanrz3@mail.sysu.edu.cn (R.Z.); zhaop36@mail2.sysu.edu.cn (P.Z.); lbaoh@mail.sysu.edu.cn (B.L.)

* Correspondence: stszhyu@mail.sysu.edu.cn (Y.Z.); stsdsz@mail.sysu.edu.cn (S.D.)

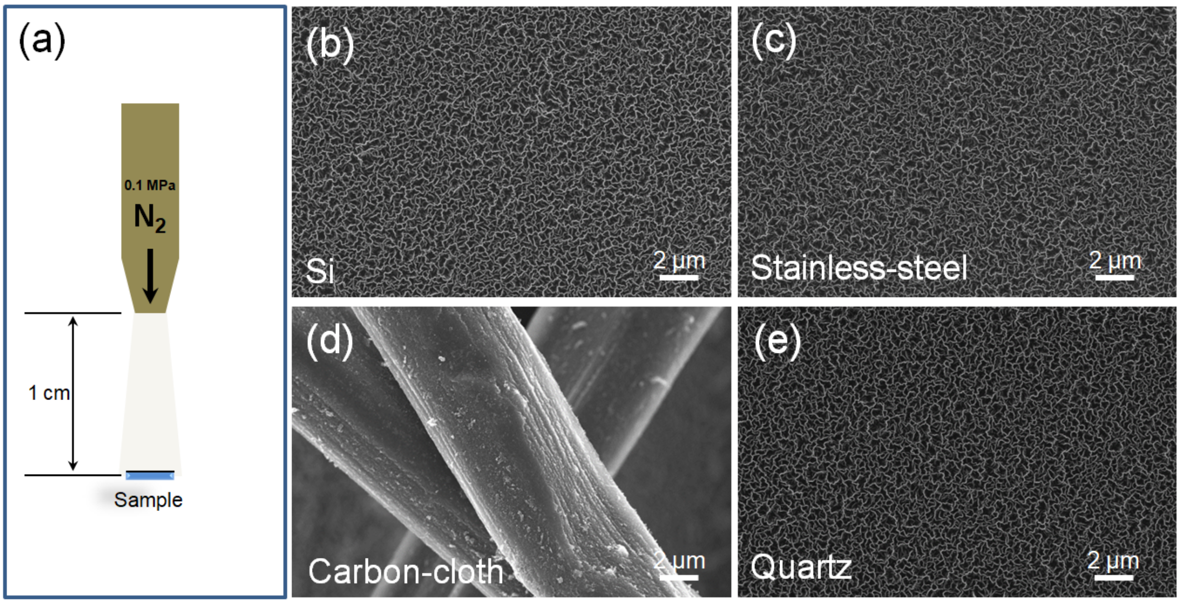

Figure S1. (**a**) Adhesion test of VFLG on different substrates. (**b–e**) SEM images of VFLG on four substrates after blowing N_2, where (**b**) Si substrate, (**c**) stainless-steel substrate, (**d**) flexible carbon-cloth substrate, and (**e**) quartz substrate.

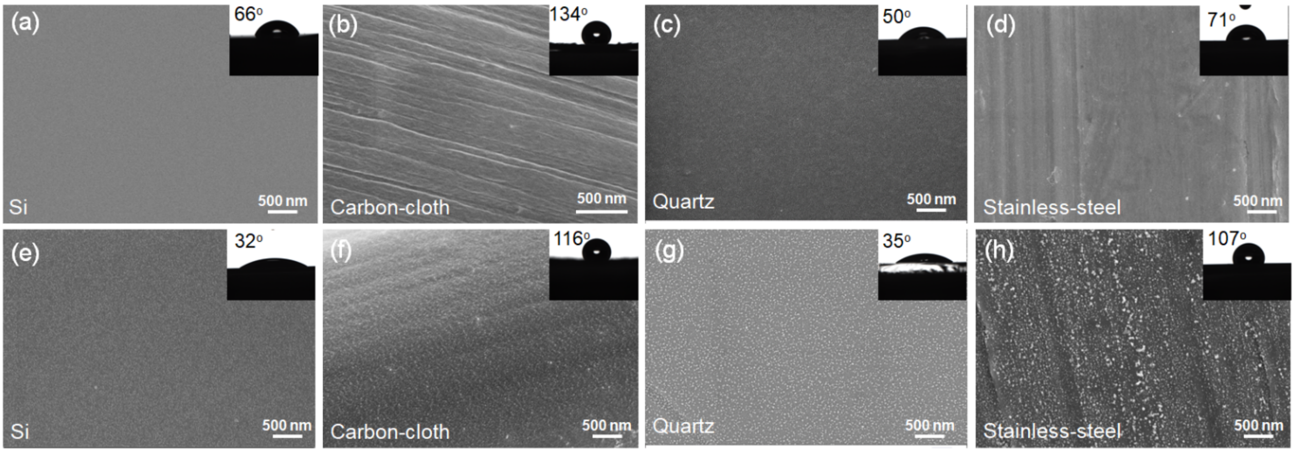

Figure S2. (**a–d**) SEM images of microscale surface morphology of the four substrates, where (**a**) Si substrate, (**b**) flexible carbon-cloth substrate, (**c**) quartz substrate and (**d**) stainless-steel substrate. (**e–h**) SEM images of microscale on the surface of substrates after the plasma bombardment pretreatment, where (**e**) Si substrate, (**f**) flexible carbon-cloth substrate, (**g**) quartz substrate and (**h**) stainless-steel substrate. Inserts are the contact angle which were tested using water as the medium dripped on the surface of substrates.

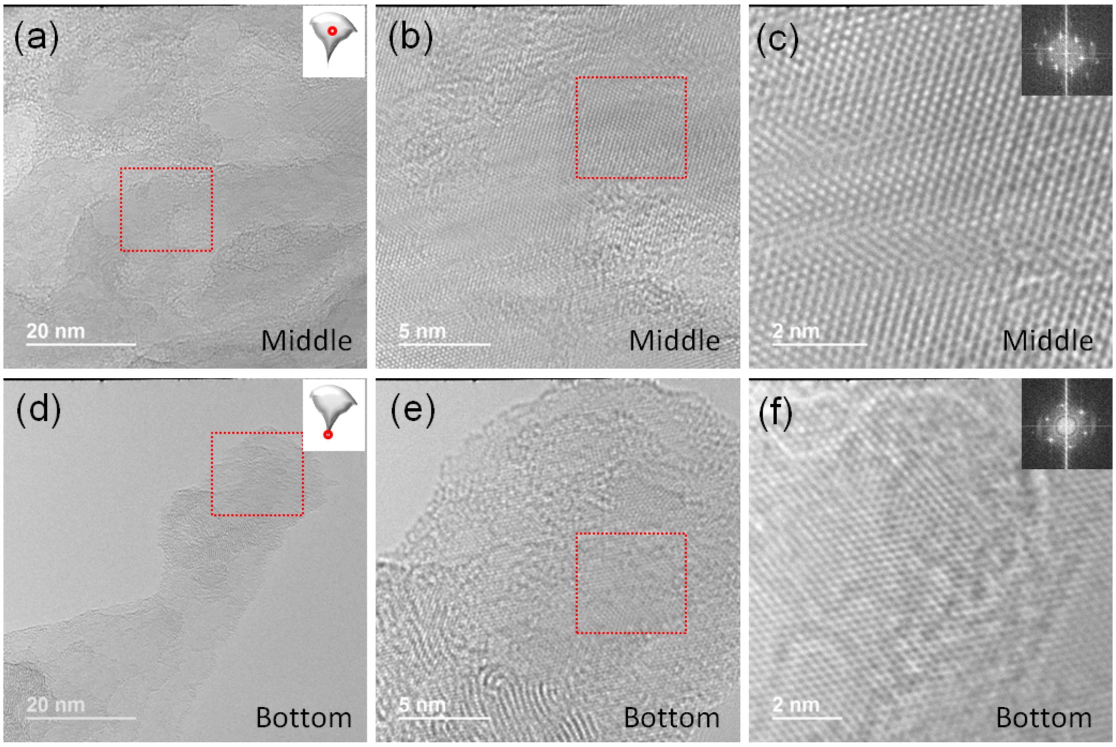

Figure S3. HRTEM images of a single VFLG sheet grown on Si substrates. (**a**) Middle of the single VFLG, (**b**) magnified image of the red rectangle area in (**a**), (**c**) HRTEM image of the red rectangle area in (**b**). (**d**) Bottom of the single VFLG, (**e**) magnified image of the red rectangle area in (**d**), (**f**) HRTEM image of the red rectangle area in (**e**). Insets of (**a**) and (**d**) show the position of HRTEM images on the VFLG sheet. Insets of (**c**) and (**f**) show the diffraction pattern of VFLG.

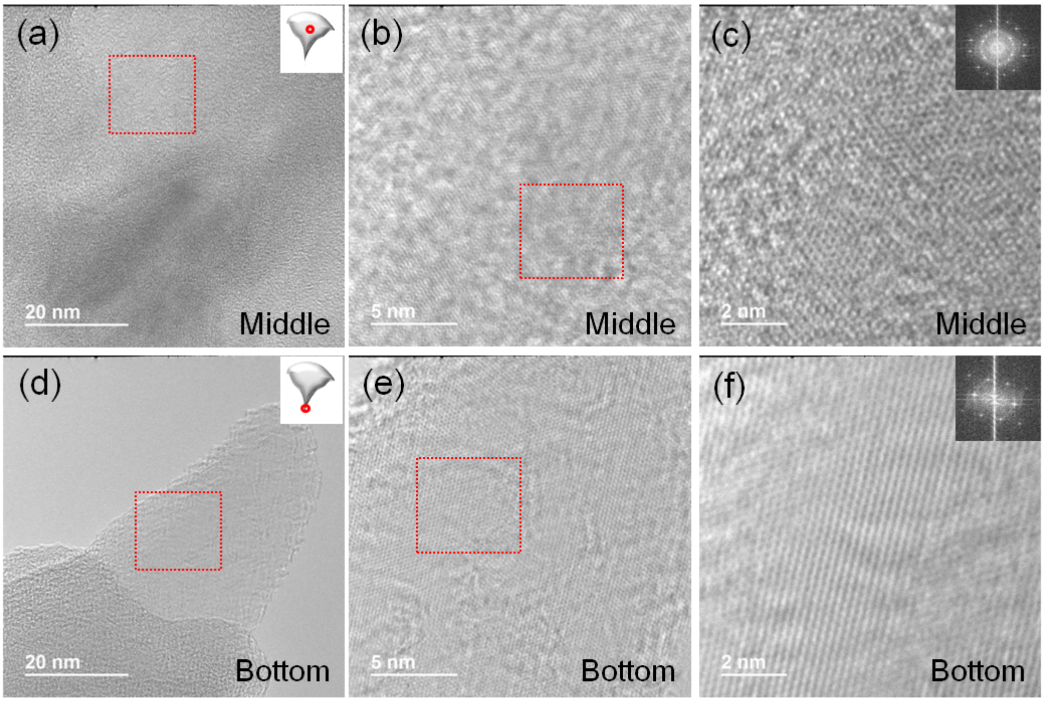

Figure S4. HRTEM images of a single VFLG sheet grown on stainless-steel substrates. (**a**) Middle of the single VFLG, (**b**) magnified image of the red rectangle area in (**a**), (**c**) HRTEM image of the red rectangle area in (**b**). (**d**) Bottom of the single VFLG, (**e**) magnified image of the red rectangle area in (**d**), (**f**) HRTEM image of the red rectangle area in (**e**). Insets of (**a**) and (**d**) show the position of HRTEM images on the VFLG sheet. Insets of (**c**) and (**f**) show the diffraction pattern of VFLG.

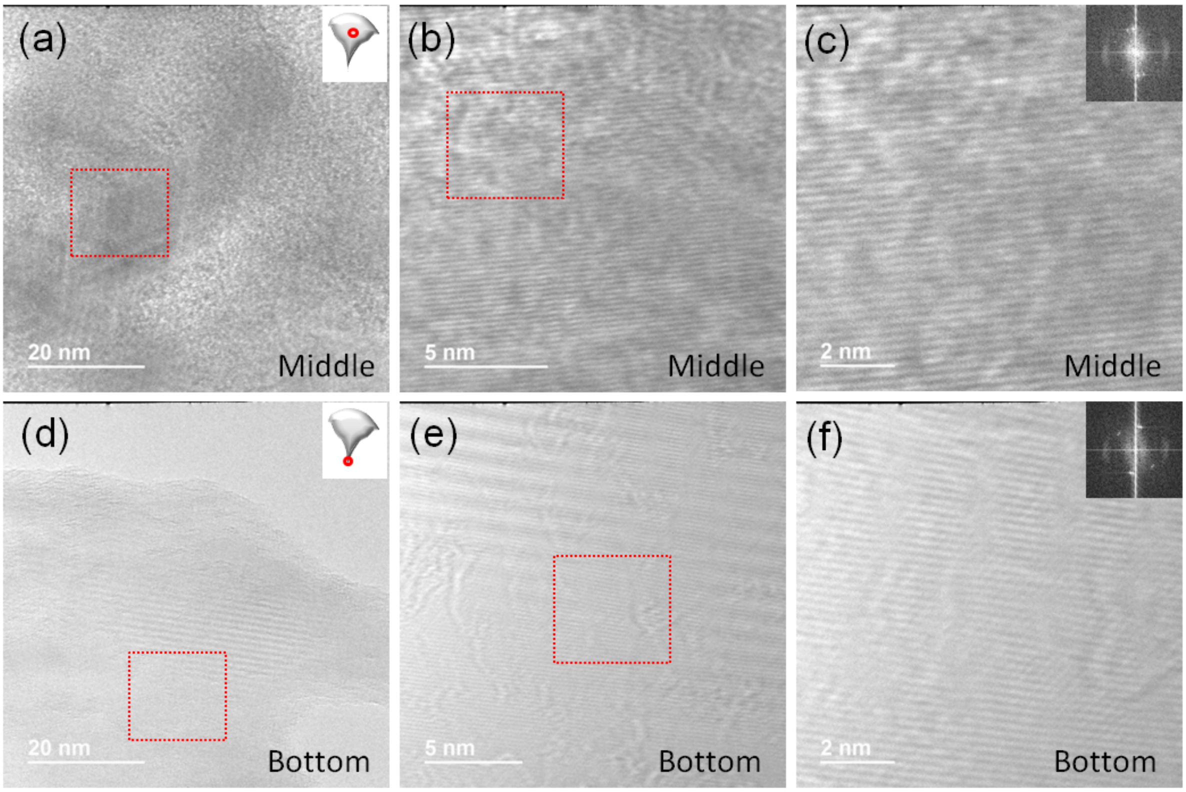

Figure S5. HRTEM images of single VFLG sheet grown on flexible carbon-cloth substrates. (**a**) Middle of the single VFLG, (**b**) magnified image of the red rectangle area in (**a**), (**c**) HRTEM image of the red rectangle area in (**b**). (**d**) Bottom of the single VFLG, (**e**) magnified image of the red rectangle area in (**d**), (**f**) HRTEM image of the red rectangle area in (**e**). Insets of (**a**) and (**d**) show the position of HRTEM images on the VFLG sheet. Insets of (**c**) and (**f**) show the diffraction pattern of VFLG.

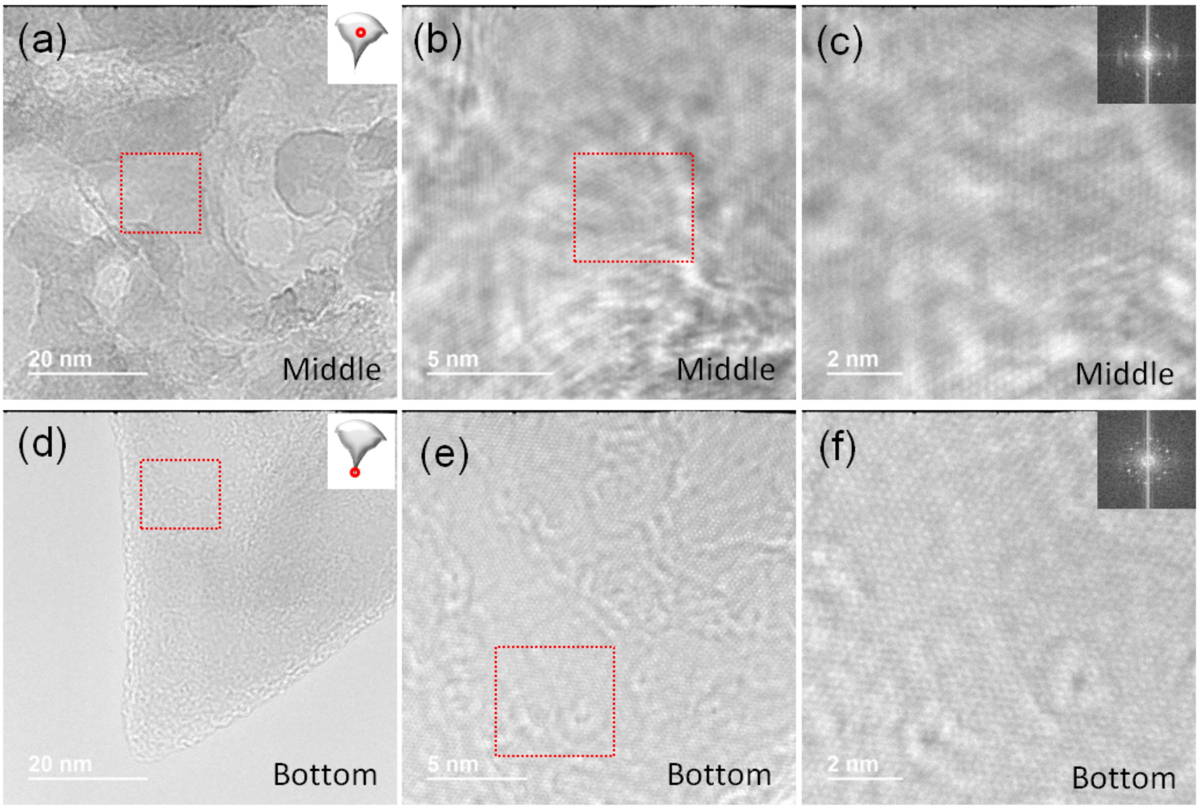

Figure S6. HRTEM images of single VFLG sheet grown on quartz substrates. (**a**) Middle of the single VFLG, (**b**) magnified image of the red rectangle area in (a), (c) HRTEM image of the red rectangle area in (**b**). (**d**) Bottom of the single VFLG, (**e**) magnified image of the red rectangle area in (**d**), (**f**) HRTEM image of the red rectangle area in (**e**). Insets of (**a**) and (**d**) show the position of HRTEM images on the VFLG sheet. Insets of (**c**) and (**f**) show the diffraction pattern of VFLG.

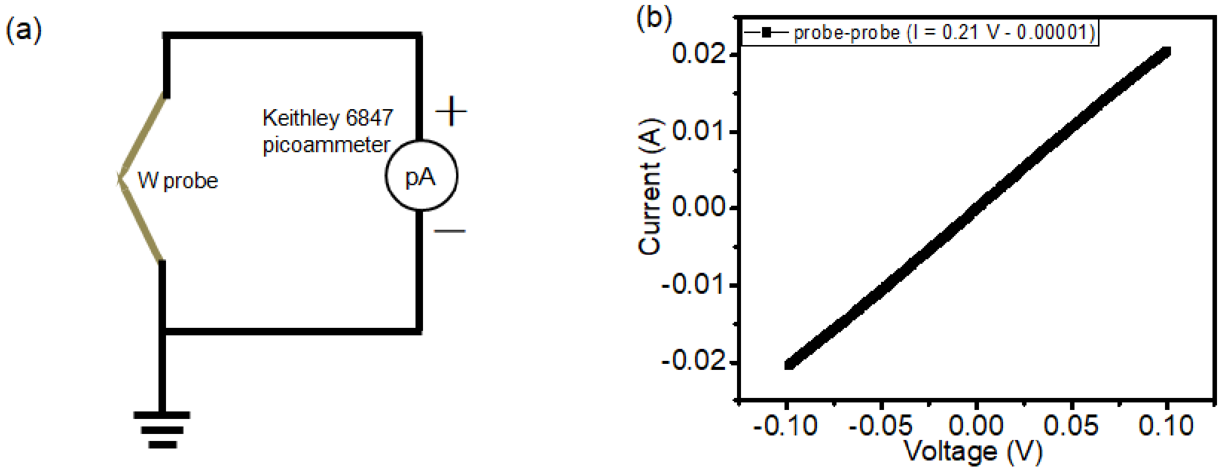

Figure S7. (**a**) The electrical test circuit of W probe to W probe test, and (**b**) the *I-V* curve of the W probe to W probe test.

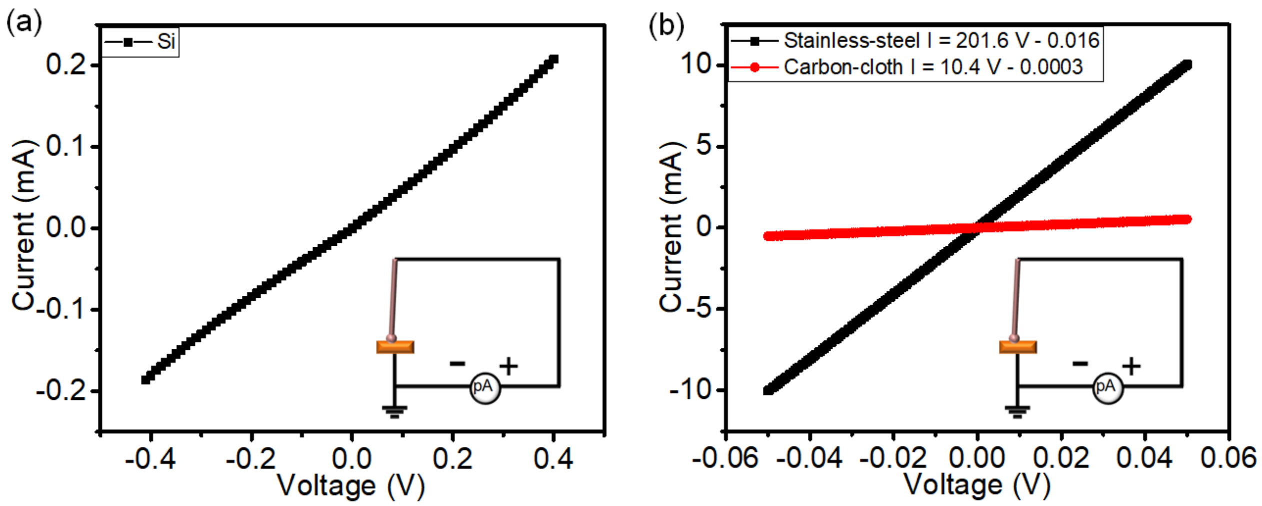

Figure S8. (**a**) *I-V* curve of the W probe to the Si substrate, (**b**) *I-V* curve of W probe to the stainless-steel and flexible carbon-cloth substrate. Insert is the test circuit diagram.

Table S1. Thermal conductivity and electrical conductivity of the Si and stainless-steel substrates.

Substrate	Thermal conductivity (W/m K)	Electrical conductivity (S/m)
n-Si	133 [1]	1.56×10^{-3} [2]
Stainless-steel	14 [3]	9×10^{6} [4]

Reference

1. J.H. Kim, A. Feldman, D. Novotny. Application of the Three Omega Thermal Conductivity Measurement Method to a Film on a Substrate of Finite Thickness, J. Appl. Phys. 1999, 86, 3959-3963.
2. D. A. Tsukanov, S. V. Ryzhkov, I. A. Be1ous, O. A. Utas, V. G. Lifshits. Electrical Conductivity of Surface Phases on Silicon. Proc. of SPIE. 2003, 5129, 295-304.
3. R. S. Graves, T. G. Kollie, D. L. Mcelroy, K. E. Gilchrist. The Thermal Conductivity of AISI 304 L Stainless Steel. Int. J. Thermophys. 1991, 12, 409-415.
4. Y. Tian. Electrical Conductivity and Magnetic Permeability Measurement of Case Hardened Steels. AIP Conf. Proc. 2015, 1650, 462-469.
